# Persian version of brief infant sleep questionnaire (BISQ): a psychometric evaluation

**DOI:** 10.1186/s12887-024-04666-6

**Published:** 2024-03-15

**Authors:** Maryam Yazdi, Maryam Bemanalizadeh, Roya Kelishadi

**Affiliations:** Child Growth and Development Research Center, Research Institute for Primordial Prevention of Non-Communicable Disease, Isfahan University of Medical Science, Isfahan, Iran

**Keywords:** Sleep, Infant, Questionnaire, Validity, Reliability, Psychometric properties, Instrument development, Persian, Brief infant sleep questionnaire, BISQ

## Abstract

**Background:**

The high prevalence of sleep problems and their negative consequences on children and parents highlight the need to design early screening instruments to evaluate sleep problems in early childhood. We aimed to determine the validity and reliability of the Brief Infant Sleep Questionnaire (BISQ) among the Iranian population.

**Methods and materials:**

This study included 646 one-year-old infants by random sampling from the PERSIAN birth cohort study. Following the forward-backward translation of the BISQ, its psychometric properties, including construct validity in terms of concurrent and convergent validities as well as reliability, were evaluated.

**Results:**

The CVIs and CVR ranged between 0.8 and 1.00 for all items. Therefore, we keep all the items of the original version of the BISQ in the Persian BISQ. Concurrent validity was assessed by comparing items of the Persian BISQ among different maternal views regarding their infant’s sleep. All BISQ items were significantly different among the two levels of maternal view about the infant’s sleep problem except daytime sleep duration. The convergent validity of the BISQ was evaluated by calculating the correlation between BISQ items and the ISQ (infant sleep questionnaire) total score as a similar tool. ISQ score was adequately correlated with nocturnal sleep latency and the number of waking at night (r_s_ ranged from 0.59 to 0.72). In addition, the associations of mothers’ and infants’ demographic variables and nutritional and gestational variables with BISQ items were presented to confirm construct validity. Strong correlations were found between the repeated sleep measures for sleep arrangement, sleep position, and sleep situation (kappa ranged from 0.65 to 0.84), nocturnal sleep duration, daytime sleep duration number of wakings at night, night waking duration, nocturnal sleep latency and sleep-onset time (ICC ranged 0.91 to 0.99).

**Conclusion:**

The Persian version of the BISQ is a reliable and valid measure for assessing sleep problems in infants. It would be helpful to be utilized for the early diagnosis of infants’ sleep problems.

**Supplementary Information:**

The online version contains supplementary material available at 10.1186/s12887-024-04666-6.

## Introduction

Infant sleep problems, which are associated with the child’s mental and physical health, are one of the most common complaints presented to pediatricians or other child-care professionals [1–3]. It has been shown that sleep problems in early childhood vary between 20 and 75% based on parents’ reports in different populations [4–6]. Fragmented sleep, night waking, inadequate sleep, and prolonged sleep onset latency are among the most common sleep problems in early childhood [5, 7]. The high prevalence of sleep problems, their negative consequences on infants’ health, and the high success ratio of clinical and educational interventions highlight the necessity for early screening to assess sleep problems during the first 3 years of life.

On the one hand, sleep assessment in early childhood has always faced some challenges in epidemiological investigations. Polysomnography, as the gold standard for assessing sleep disturbances, and actigraphy, as an alternative to polysomnography, are still limited since they require expensive devices and complex assessments [8, 9]. On the other hand, a large proportion of physicians do not routinely screen infants and toddlers for sleep problems [2]. In addition, it is estimated that only 46% of the pediatricians felt confident in their ability to identify sleep problems. Evidence indicates that additional educational opportunities are needed in pediatric sleep medicine in medical schools. Moreover, a recent study found that sleep problems are rarely addressed in general pediatric clinics prior to their identification by parental responses on a validated sleep questionnaire [35]. Thus, the availability of a brief validated screening tool would facilitate regular professional screening. A screening tool that is also accessible to parents who are concerned about their child’s sleep can facilitate early routine professional screenings. The use of a standardized, brief, valid questionnaire for infants’ sleep assessment has never been established in the Persian language. The Brief Infant Sleep Questionnaire (BISQ) is a screening instrument for daytime and nighttime routines and sleep patterns in early childhood based on parental responses [10].

Sleep differences among populations are a cross-cultural phenomenon, and most existing studies have been limited to Western populations, not the Middle East of Asia. Considering the key role of sleep in children’s health, a standardized sleep questionnaire for infants for use in pediatric practices and research has been increasingly valued. We aimed to develop a valid and reliable questionnaire in the Persian version for assessing sleep behaviors in infants. The aims of the present study were (1) to translate the BISQ into Persian and (2) to determine the validity and reliability of the BISQ for the Persian population.

## Methods and materials

### Study design and participants

This survey was conducted between November 2020 and February 2022 on a random subsample of infant/parent pairs from the ongoing PERSIAN birth cohort study in Isfahan including a total of 3000 mother/infant pairs at the initiation of this study [11]. The 12-month infant/parent pairs were selected using random sampling stratified by gender groups. Infants under treatment with medications related to sleep and those with a history of admission to neonatal intensive care units were excluded from our study. The parents were contacted on the phone calls and were asked about their time availability to confirm the date of the interview.

All data, including the BISQ, the checklist of demographic characteristics, and other related outcomes, were collected through phone calls by our interviewers and concurrently were recorded online through Google Forms. All parents received enough information about the study, and oral informed consent was obtained from them. Finally, 646 mother/infant pairs were recruited for the study.

### Brief infant sleep questionnaire (BISQ)

In 2004, the BISQ was developed by Sadeh to screen sleep patterns in infants and toddlers (0–3 years) [10]. The items of the BISQ include nocturnal sleep duration (between the hours of 7 pm and 7 am); daytime sleep duration (between the hours of 7 am and 7 pm); number of night wakes; duration of wakefulness during the night hours (10 pm to 6 am); nocturnal sleep-onset time (the clock time at which the child falls asleep for the night); settling time (latency to falling asleep for the night); method of falling asleep; location of sleep; preferred body position; and the following general information: age of child; gender of child; birth order; and role of responder (who completed the BISQ). The administration time of the questionnaire is 5 to 10 min, and questions are related to the last week’s sleep periods of the infants. The criteria used to define poor sleepers according to the BISQ measures are as follows: (1) the child wakings > 3 times per night; (2) the nocturnal wakefulness period > 1 h; or (3) the total sleep time < 9 h.

### Other measurements

A comprehensive checklist was used to collect information including infants’ gender, gestational age, birth order, nutritional status during the first year of life, delivery type, maternal age, maternal education, maternal job, and marital status.

### Procedures

#### Translation

The BISQ was translated from English to Persian using the methodology given by Beaton et al. [12]. Two independent professional translators translated it into Persian. One of them was familiar with the concept of the items being translated, whereas the other was unaware of the items being examined in the original English questionnaire. Then, the current study’s researchers (MB and MY) and both translators adopted a consolidated forward version (forward translation). This questionnaire was then backward translated into English by two bilingual translators to compare with the original one to ensure conceptual equivalence (backward translation). Following a careful review by researchers (MB and MY), needed revisions were made, and the provisional Persian version of the BISQ was provided. In general, there were no difficulties with the translated questionnaire.

#### Content and face validity

We determined content validity using the content validity index (CVI) and content validity ratio (CVR). The CVI measures the simplicity, relevance, and clarity of each question with the construct evaluated by the scale. We asked ten specialists (4 pediatricians, 4 epidemiologists, and biostatisticians with working experience in the field of infants and toddlers and 2 infant caregivers) to rate the simplicity, relevance, and clarity of the Persian BISQ questions on a 4-point rating scale. For example, the experts assessed the relevance of the items using [1] irrelevant; [2] slightly relevant; [3] relevant; and [4] completely relevant. A CVI of ≥ 0.79 was considered acceptable for each question [13, 14]. The CVR assesses the necessity of each question. For calculating CVR, experts were asked to rate the essentiality of the Persian BISQ questions on a 3-point rating scale, i.e., [1] unnecessary; [2] useful but unnecessary; and [3] necessary. A CVR of ≥ 0.62 was considered satisfactory for each rating [13, 14]. Qualitative face validity involved the expert panel and parents who evaluated the BISQ for difficulty, relevance, and ambiguity. Then, the Persian version of the BISQ was finalized and used for evaluating the psychometric properties.

#### Psychometric analysis of the BISQ

In this study, psychometric properties of the BISQ, including validity (concurrent and convergent validity) and reliability (test-retest reliability and internal consistency), were evaluated. The quantitative and qualitative variables were expressed as the mean ± standard deviation (SD) and number (percentage), respectively. Data analyses were performed using SPSS (version 23; SPSS Inc., Chicago, IL, USA).

#### Concurrent validity

Concurrent validity was investigated by the BISQ’s ability to discriminate between healthy infants and infants with sleep problems based on maternal view. We expected that the sleep pattern of an infant with sleep problems defined by the maternal view would be significantly different from that of an infant without it. The validity of the measure is supported if the distribution of each item is significantly different among the groups. We tested differences among the groups (healthy infants and infants having sleep problems according to maternal view) using the Mann‒Whitney test.

#### Convergent validity

The convergent validity of the BISQ total score was evaluated by calculating the correlation between BISQ items and the ISQ (infant sleep questionnaire) total score as a similar tool. ISQ is a well-published, highly validated measure that was developed by Morrell [15]. It was developed as a screening tool for sleep problems. It is a parent-reported questionnaire designed specifically to assess sleeping behaviors in 12- to 18-month-old infants. This parent-reported questionnaire includes specific questions about daytime and nighttime sleep patterns, as well as sleep-related behaviors over the last month. It has 9 main questions regarding infants’ sleep. The ISQ score ranged between 0 and 38. Higher scores indicate more severe sleep problems. Spearman correlation (r_s_) was reported because of departure from normality in ISQ score. r_s_< 0.2 was considered as week correlation and r_s_>0.5 as adequate correlation.

Furthermore, the associations of mothers’ and infants’ demographic variables and nutritional and gestational variables with BISQ items were presented in order to confirming construct validity.

#### Reliability

We recruited 30 infants to investigate internal consistency and test-retest reliability. The parents were asked to participate in two interviews on two separate days at 2-week intervals. All interviews were conducted on phone calls and performed by trained interviewers. To determine test-retest reliability, for quantitative items, the intraclass correlation coefficient (ICC), along with 95% confidence, was computed using a two-way mixed model. A coefficient of more than 0.70 was considered excellent stability [16]. For qualitative items, we computed kappa measure agreement as a measure of stability of responses.

## Results

### Participant characteristics

A total of 646 infants were recruited for this study. Table 1 shows the distribution of some demographic and health-related characteristics of the participants. A total of 51.9% of infants were boys, and only 7.8% of infants were preterm (< 37 weeks). A total of 56.8% of infants were born by C-sections. We also provided some information regarding birth order, the first and second 6-month nutrition of infants and maternal characteristics in detail in Table 1.

**Table 1:** Demographic and health-related characteristics of the participants

Characteristics	Mean ± SD or Number (%)
**Infant gender**	
Boy	335(51.9)
Girl	311(48.1)
**Gestational age**	
< 37 week	50(7.7)
≥ 37 weeks	596(92.3)
**Birth order**	
Firstborn	299(46.3)
Second born	273(42.3)
Third born	59(9.1)
Fourth born	13(2.0)
Fifth-born or more	2(0.3)
**First 6 months of nutrition**	
Breastfeeding	451(69.8)
Formula	14(2.2)
Breastfeeding + formula	181(28.0)
**Second 6 months of nutrition**	
Breastfeeding	5(0.8)
Breastfeeding + formula	1(0.2)
Breastfeeding + food	470(72.8)
Formula + food	76(11.8)
Breastfeeding + formula + food	94(14.6)
**Maternal age (years)**	30.78 ± 5.19
**Maternal job status**	
unemployed	593(91.8)
employed	53 (8.2)
**Maternal education**	
Under diploma	90(13.9)
Diploma	240(37.2)
Upper diploma	316(48.9)
**Marital status**	
Married	644(99.7)
Separated/divorced	2(0.3)
**Delivery type**	
Normal Vaginal delivery	281(43.5)
C-section	365(56.5)

### Content and face validity

The panel of experts examined the relevance, simplicity, and clarity of the wording and phrasing of the Persian BISQ questions. The CVIs and CVR ranged between 0.8 and 1.00 for all questions. Therefore, we keep all the questions of the original version of the BISQ in the Persian BISQ (Table 2).

**Table 2:** Relevance, simplicity, clarity, I-CVI, and CVR values of the Persian BISQ

Items	Questions	CVI			I-CVI	CVR
		Relevance	Simplicity	Clarity		
Sleeping arrangement	Sleeping arrangement (infant crib in a separate room, infant in crib in parents’ room, in parent’s bed, infant crib in room with sibling)	0.9	0.8	0.8	0.83	0.8
Sleep position	In what position does your child sleep most of the time? (on his/her belly, on his/her side, on his/her back)	0.8	0.8	0.9	0.83	0.8
Nocturnal sleep duration (hour)	How much time does your child spend in sleep during the NIGHT (between 7 in the evening and 7 in the morning)? (hour)	1	0.9	1	0.97	1
Daytime sleep duration (hour)	How much time does your child spend in sleep during the DAY (between 7 in the morning and 7 in the evening)? (hour)	1	0.9	1	0.97	1
Number of waking at night	Average number of night wakings per night	1	1	1	1.00	1
Night waking duration (hour)	How much time during the night does your child spend in wakefulness (from 10 in the evening to 6 in the morning)? (hour)	1	0.8	0.9	0.90	1
Nocturnal sleep latency (hour)	How long does it take to put your baby to sleep in the evening? (hour)	1	1	1	1.00	1
Sleep situation	How does your baby fall asleep? (while feeding, being rocked, being held, in bed alone, in bed near parent)	0.8	0.8	0.8	0.80	0.8
Sleep onset time (hour: minute)	When does your baby usually fall asleep for the night? (hour: minute)	1	1	1	1.00	1
Maternal view	Do you consider your child’s sleep as a problem?	1	1	0.9	0.97	1

*Abbreviations* BISQ: Brief Infant Sleep Questionnaire; CVI: Content validity index; I-CVI: Item-content validity index; CVR: Content validity ratio

Table 3 shows the distribution of BISQ items among the participants. A total of 95.4% of infants slept in the parents’ room or parents’ bed. The most common position (50%) of infants during sleep was on his/her back. Moreover, most of them (82.7%) slept while feeding. The mean sleep onset of infants was 11.26 ± 1.07 (h). The mean nocturnal sleep duration, daytime sleep duration, night waking duration and nocturnal sleep latency were 7.71 ± 1.28, 3.81 ± 1.63, 1.31 ± 1.13, and 0.32 ± 0.23 (h), respectively.

**Table 3:** Distribution of Persian BISQ items within the scoring system among the infants

Items	Number (%) or Mean ± SD
**Sleep arrangement**	
Infant crib in separate room	23(3.6)
Infant in crib in parents’ room	327(50.6)
In parent’s bed	293(45.4)
Infant crib in room with sibling	3(0.5)
**Sleep position**	
On his/her belly	211(32.7)
On his/her side	112(17.3)
On his/her back	323(50.0)
**Sleep situation**	
While feeding	534(82.7)
Being rocked	78(12.1)
Being held	12(1.9)
In bed alone	17(2.6)
In bed near parent	5(0.8)
**Sleep onset time (hour: minute)**	11.26 ± 1.07
**Nocturnal sleep duration (hour)**	7.71 ± 1.28
**Daytime sleep duration (hour)**	3.81 ± 1.63
**Night waking duration (hour)**	1.31 ± 1.13
**Nocturnal sleep latency (hour)**	0.32 ± 0.23
**Parents’ perception**	
Not a problem	568(87.9)
A small problem	77(11.9)
A very serious problem	1(0.2)

*Abbreviations* BISQ: Brief Infant Sleep Questionnaire; IQR: Interquartile range

### Concurrent validity

We presented the results of the concurrent validity of the Persian BISQ based on comparing quantitative BISQ items among different maternal views. For instance, the mean ± SD of nocturnal sleep duration in groups of infants with no sleep problems and with mild to moderate sleep problems based on the maternal view were 7.78 ± 1.26 and 7.16 ± 1.26 (h), respectively (*P* < 0.001). All items were significantly different among the two levels of maternal view about the infant’s sleep problem except daytime sleep duration (Table 4).

**Table 4:** Comparison of the BISQ items among infants with or without sleep problems based on maternal view

Items	Maternal view		p ^a^
	With no sleep problem (*n* = 568)	With mild to severe sleep problems (*n* = 78)	
Nocturnal sleep duration (hour)	7.78 ± 1.26^b^	7.16 ± 1.26	< 0.001
Daytime sleep duration (hour)	3.79 ± 1.63	3.97 ± 1.64	0.475
Number of waking at night	0.72 ± 1.19	2.12 ± 1.80	< 0.001
Night waking duration (hour)	1.22 ± 1.07	1.96 ± 1.32	< 0.001
Nocturnal sleep latency (hour)	0.30 ± 0.22	0.43 ± 0.28	< 0.001
Sleep onset time (hour)	23.19 ± 1.02	23.74 ± 1.30	< 0.001

*Abbreviations* BISQ: Brief Infant Sleep Questionnaire

^a^ Calculated by the Mann‒Whitney test

^b^ Mean ± SD

### Convergent validity

The convergent validity of the BISQ was evaluated by calculating the correlation between BISQ items and the ISQ total score as a similar tool. Table 5 shows Spearman’s correlation between quantitative items of the BISQ and ISQ total score.

**Table 5:** The association of BISQ quantitative items with ISQ total score

Items	Correlation with ISQ total score	p
	r_s_	
Nocturnal sleep duration (hour)	-0.147	< 0.001
Daytime sleep duration (hour)	-0.112	0.004
Number of waking at night	0.719	< 0.001
Night waking duration (hour)	0.221	< 0.001
Nocturnal sleep latency (hour)	0.589	< 0.001
Sleep onset time (hour)	0.133	0.001

*Abbreviations* ISQ: infant sleep questionnaire, r_s_: Spearman correlation

Additionally, the association of demographic, nutritional, and gestational variables with BISQ items is presented in Table 6. Daytime sleep duration was significantly higher among children who were nourished by both breastfeeding and formula. The mean nocturnal sleep latency was the lowest among infants who were nourished by food plus breastfeeding in their second 6 months, while in infants without complementary food, it was highest (0.30 ± 0.20 vs. 0.44 ± 0.35 (hour), *p* = 0.003). There was a significant inverse association between birth order and infants’ better sleep. Nocturnal sleep duration was less reported in firstborns, while night waking duration, daytime sleep duration, and nocturnal sleep latency were higher among them (*p* < 0.05). Furthermore, the firstborn infants’ sleep onset time was later than that of the second- and higher-order-born infants (*p* = 0.007). Infant’s number of waking at night was higher among those with older (*p* = 0.015) and more educated mothers (*p* = 0.033). The term infants had significantly shorter night waking duration (*p* = 0.036) and lower sleep latency (*p* = 0.02).

**Table 6:** Association of demographic, nutritional, and gestational variables with BISQ items

	Nocturnal sleep duration (hour)	Daytime sleep duration (hour)	Number of waking at night	Night wakings duration (hour)	Nocturnal sleep latency (hour)	Sleep onset time (hour)
**First 6 months of nutrition**						
Breastfeeding	7.73 ± 1.25	3.71 ± 1.64	0.95 ± 1.38	1.27 ± 1.11	0.30 ± 0.20	23.25 ± 1.07
Formula	8.07 ± 1.58	3.64 ± 1.51	1.00 ± 1.36	1.54 ± 1.20	0.43 ± 0.44	23.68 ± 0.50
Breastfeeding + formula	7.62 ± 1.32	4.08 ± 1.60	0.73 ± 1.26	1.37 ± 1.16	0.36 ± 0.26	23.26 ± 1.10
p	0.501	0.036	0.123	0.493	0.069	0.131
**Second 6 months of nutrition**						
Breastfeeding + food	7.74 ± 1.23	3.73 ± 1.67	0.92 ± 1.36	1.27 ± 1.11	0.30 ± 0.20	23.25 ± 1.09
Breastfeeding + formula	7.64 ± 1.59	4.06 ± 1.65	0.91 ± 1.37	1.48 ± 1.24	0.44 ± 0.35	23.27 ± 1.20
Breastfeeding + formula + food	7.58 ± 1.25	4.02 ± 1.35	0.72 ± 1.29	1.37 ± 1.09	0.33 ± 0.21	23.31 ± 0.83
p	0.245	0.045	0.266	0.297	0.003	0.425
**Birth order**						
firstborn	7.56 ± 1.32	4.06 ± 1.65	0.90 ± 1.38	1.47 ± 1.17	0.34 ± 0.22	23.38 ± 1.13
second born	7.84 ± 1.23	3.60 ± 1.58	0.96 ± 1.40	1.20 ± 1.07	0.31 ± 0.24	23.18 ± 1.00
third and higher-born	7.80 ± 1.25	3.61 ± 1.61	0.58 ± 0.99	1.00 ± 1.05	0.26 ± 0.18	23.06 ± 1.06
p	0.018	0.002	0.280	0.001	0.005	0.007
**Maternal age**						
≤ 30	7.70 ± 1.22	3.76 ± 1.70	1.02 ± 1.43	1.34 ± 1.07	0.34 ± 0.22	23.28 ± 1.02
> 30	7.71 ± 1.33	3.87 ± 1.56	0.77 ± 1.26	1.27 ± 1.18	0.30 ± 0.23	23.24 ± 1.12
p	0.928	0.206	0.015	0.245	0.002	0.637
**Maternal education**						
Under diploma	7.88 ± 1.25	3.44 ± 1.66	1.11 ± 1.37	1.09 ± 1.03	0.31 ± 0.24	23.11 ± 1.06
Diploma	7.64 ± 1.20	3.83 ± 1.68	0.85 ± 1.30	1.30 ± 1.10	0.32 ± 0.23	23.25 ± 1.09
Upper diploma	7.70 ± 1.34	3.91 ± 1.57	0.85 ± 1.38	1.37 ± 1.17	0.32 ± 0.22	23.31 ± 1.05
p	0.188	0.067	0.033	0.134	0.828	0.154
**Maternal job status**						
unemployed	7.70 ± 1.28	3.79 ± 1.63	0.90 ± 1.35	1.30 ± 1.11	0.32 ± 0.22	23.24 ± 1.06
employed	7.71 ± 1.23	4.03 ± 1.66	0.72 ± 1.34	1.42 ± 1.25	0.34 ± 0.29	23.43 ± 1.13
p	0.853	0.240	0.245	0.694	0.971	0.248
**Infant gender**						
Boy	7.82 ± 1.19	3.79 ± 1.70	0.88 ± 1.34	1.25 ± 1.07	0.31 ± 0.22	23.19 ± 1.02
Girl	7.58 ± 1.36	3.84 ± 1.55	0.90 ± 1.37	1.36 ± 1.18	0.32 ± 0.24	23.34 ± 1.12
p	0.042	0.568	0.855	0.395	0.624	0.204
**Delivery type**						
Normal Vaginal delivery	7.67 ± 1.27	3.76 ± 1.64	0.98 ± 1.42	1.32 ± 1.13	0.33 ± 0.24	23.24 ± 1.01
C-section	7.73 ± 1.29	3.86 ± 1.62	0.82 ± 1.30	1.30 ± 1.13	0.31 ± 0.22	23.27 ± 1.11
p	0.687	0.338	0.108	0.814	0.676	0.733
**Gestational age**						
< 37 week	7.63 ± 1.55	3.48 ± 1.90	0.86 ± 1.37	1.61 ± 1.11	0.42 ± 0.33	23.36 ± 0.95
≥ 37 weeks	7.71 ± 1.25	3.84 ± 1.60	0.89 ± 1.35	1.28 ± 1.12	0.31 ± 0.22	23.25 ± 1.08
p	0.446	0.192	0.853	0.036	0.020	0.211

### Reliability

Strong correlations were found between the repeated sleep measures for sleep arrangement (kappa = 0.65), sleep position (kappa = 0.84), sleep situation (kappa = 0.66), nocturnal sleep duration (r_s_ =0.91, ICC = 0.92), daytime sleep duration (r_s_ =0.91, ICC = 0.94), number of wakings at night (r_s_ =0.96, ICC = 0.96), night wakening duration (r_s_ =0.89, ICC = 0.97), nocturnal sleep latency (r_s_ =0.98, ICC = 0.98) and sleep-onset time (r_s_ =0.93, ICC = 0.99). All correlations were significant with *p* < 0.001 (See Table 7).

**Table 7:** Reliability statistics of the Persian BISQ

Items	kappa	ICC	(95% CI)
Sleeping arrangement	0.65	-	-
Sleep position	0.84	-	-
Sleep situation	0.66	-	-
Nocturnal sleep duration (hour)	-	0.92	(0.84,0.96)
Daytime sleep duration (hour)	-	0.94	(0.87,0.97)
Number of waking at night	-	0.96	(0.92,0.98)
Night waking duration (hour)	-	0.97	(0.94,0.99)
Nocturnal sleep latency (hour)	-	0.98	(0.96,0.99)
Sleep onset time (hour: minute)	-	0.99	(0.99,1.00)

*Abbreviations* BISQ: Brief Infant Sleep Questionnaire; ICC: Intra class coefficient; CI: Confidence interval

All ICCs were significant at the *P* < 0.001 level

## Discussion

In the current study, the psychometric properties of the Persian version of BISQ were examined. The BISQ is a standardized screening tool for evaluating infants’ sleep that was developed by Sadeh in 2004 [10]. It is a parent-reported questionnaire designed specifically to assess sleeping behaviors in infants between 0 and 3 years of age for clinical or research purposes. Sadeh suggested several cutoffs for infants to be referred for further clinical evaluation if they meet any of the following: (1) waking more than 3 times per night, (2) wakefulness more than 1 h per night, or (3) sleep less than 9 h per 24-hour period [10].

Despite several valid child’s sleep questionnaires for the Persian-speaking population [17–21], to the best of our knowledge, the BISQ is the first Persian version of a fully validated questionnaire to measure sleep problems, particularly in infants. This questionnaire has also been validated in several languages, including English [10], Brazilian [22], Portuguese [23], Nepali [24], and Turkish [25, 26]. Del-Ponte et al. conducted a study among 586 Brazilian children and showed high specificity of the BISQ sleep parameters among their population [22].

Overall, the results of this study showed good validity of the Persian BISQ, which is consistent with previous studies demonstrating the validity of the BISQ against actigraphy [10] and daily logs [25] and its high sensitivity and specificity in documenting sleep patterns in infants [22, 27].

We found a statistically significant increase in the duration of daytime sleep among children who had a combination of breastfeeding and formula feeding. The average duration of sleep onset throughout the night was found to be significantly lower in infants who received both food and breastfeeding during their second six months of life compared to infants who did not receive complementary food. A study among 654 infants showed that despite more night awakenings, infants who were exclusively breastfed exhibited longer durations of both night sleep and total sleep when compared to those who were fed formula [28]. A notable negative correlation was observed between birth order and infants’ better sleep. One recent study has shown that the implementation of a responsive parenting intervention for first-time mothers had a positive effect on the sleep duration and behaviors of secondborn children, which is consistent with our findings [29]. Nocturnal sleep duration was less reported in firstborns, while night waking duration, daytime sleep duration, and nocturnal sleep latency were higher among them. Furthermore, the firstborn infants’ sleep onset time was later than that of the second- and higher-order-born infants. These findings are rather contradictory to previous studies. For instance, in a study among 52 infants, first-borns had fewer nocturnal awakenings and a longer consecutive nocturnal sleep duration than non-first-borns but did not differ in terms of total nocturnal sleep duration [30, 31]. Infant’s number of waking at night was higher among those with older and more educated mothers. The association between infants’ sleep patterns and some socioeconomic variables, including maternal education, has been demonstrated in current studies [32, 33]. Term infants had a significantly shorter night waking duration and lower sleep latency. Previous studies have also highlighted some differences in sleep patterns between term and preterm infants [34, 35].

Assessments of stability were derived through test-retest reliability procedures. Test-retest reliability showed significant strong correlations between repeated sleep measures in the Iranian population of infants (varied between 0.92 and 0.99). The test-retest correlations ranged between 0.82 and.95 in the original study, which was consistent with our results [10]. On the other hand, in two studies, reliability coefficients from repeated administrations of the BISQ in the Turkish population ranged from 0.35 to 0.85 [25, 26].

One of the strengths of this study is that we could recruit a large sample of infants. In addition, all the interviews were conducted one by one on phone calls, and we made sure that all mothers understood the content accurately. This method is more valid and reliable for developing standard tools [21]. However, a potential threat to this methodology could be the lack of non-verbal cues during phone calls. Phone interviews may limit the researchers’ ability to observe facial expressions and body language, which are valuable indicators of participant understanding or potential areas of confusion. Without visual cues, there’s a possibility that nuances in communication or participant responses may be overlooked, impacting the thoroughness of the assessment. To mitigate this potential threat, researchers employed additional clarifying questions or follow-ups to ensure comprehensive understanding during the phone interviews.

It should be noted that based on the literature, mothers and fathers have sometimes discrepancies in reporting children’s sleep problems. Due to the absence of data from fathers in our study, the results should be interpreted with caution. To mitigate this limitation obtaining data from both parents and using dyadic analysis are recommended [36, 37]. Future studies should consider this aspect for study design.

Our sampling was random, so it is unlikely that only motivated parents and parents of children with sleep problems participated in our study. However, since we included infants from a limited population, the representativeness of this sample for all Iranian infants or other Persian language countries should be interpreted with caution. This limitation is more prominent when we just included 1 year old children.

Although there is a revised version of the tool that was developed in 2020, at the study initiation, the revised version was not available. Thus, we employed its earlier version and continued the study with the previous version for consistency. Future studies should be conducted for assessing the validity and reliability of the BISQ-R in Persian populations. Moreover, the psychometric properties assessed are related to a sample of 12-months children. Since sleep-wake patterns change rapidly according to age, further studies on other populations of different ages should be conducted to generalize these results.

## Conclusion

The findings support the satisfactory reliability and validity of the BISQ among the Persian-speaking population. Therefore, it can be considered a standardized tool for evaluating infants’ sleep behaviors. This questionnaire can facilitate regular professional screening. It is easy to understand and takes nearly 5 min to complete. Further studies among large populations in different settings (i.e., primary health care services, hospitals, and clinics) can confirm its validity and reliability.

### Electronic supplementary material

Below is the link to the electronic supplementary material.

Supplementary Material 1

## Data Availability

All data generated or analysed during this study are included in this published article and its supplementary information files.
